# LINC01296/miR-141-3p/ZEB1-ZEB2 axis promotes tumor metastasis via enhancing epithelial-mesenchymal transition process

**DOI:** 10.7150/jca.55626

**Published:** 2021-03-05

**Authors:** Zhenqiang Sun, Bo Shao, Zaoqu Liu, Qin Dang, Yaxin Guo, Chen Chen, Yuying Guo, Zhuang Chen, Jinbo Liu, Shengyun Hu, Weitang Yuan, Quanbo Zhou

**Affiliations:** 1 Department of Colorectal Surgery, The First Affiliated Hospital of Zhengzhou University, Zhengzhou 450052, Henan, China.; 2 School of Life Sciences, Zhengzhou University, Zhengzhou 450001, Henan, China.; 3 Department of Interventional Radiology, The First Affiliated Hospital of Zhengzhou University, Zhengzhou, 450052, Henan, China.; 4 Academy of Medical Sciences, Zhengzhou University, Zhengzhou 450052, Henan, China.; 5 Department of Basic Medical, Academy of Medical Sciences of Zhengzhou University, Zhengzhou 450052, Henan, China.; 6 Henan Academy of Medical and Pharmaceutical Sciences, Zhengzhou University, Zhengzhou 450052, Henan, China.

**Keywords:** tumor metastasis, long noncoding RNA, non-small cell lung cancer, colorectal cancer, epithelial-mesenchymal transition

## Abstract

**Purpose:** Tumor metastasis seriously affects the survival of patients. In recent years, some studies confirmed that long non-coding RNA (lncRNA) played an essential role in tumor progression. A few studies reported that LINC01296 acted as an oncogenic regulator of cancer. However, its in-depth specific biological mechanism in tumor metastasis is still unknown.

**Methods:** Real-time quantitative PCR (qPCR) was performed to detect the expression of LINC01296 and miR-141-3p in NSCLC, CRC tissues and cell lines, and the dual luciferase report was used to evaluate the relationship between LINC01296, miR-141-3p and ZEB1/ZEB2 relationship. Western blot experiments are used to detect changes in protein levels. Transwell and wound healing measures migration and invasion capabilities.

**Results:** In this study, we used non-small cell lung cancer (NSCLC) and colorectal cancer (CRC) as the research objects, LINC01296 was found to be highly expressed in NSCLC and CRC tissues and positively related to poor prognosis. We also demonstrated LINC01296 regulated NSCLC and CRC invasion and metastasis by modulating epithelial-mesenchymal transition (EMT) by up-regulating ZEB1 and ZEB2. Consequently, LINC01296 acted as a sponge of miR-141-3p, which negatively regulates EMT process.

**Conclusions:** The report revealed a new mechanism by which LINC01296 regulates the EMT process through miR-141-3p/ZEB1-ZEB2 axis and affects cancer metastasis.

## Introduction

Tumor metastasis refers to the process in which malignant cells continue to grow from the original site, through lymphatic tracts, blood vessels, and other pathways to other sites. As we all know, tumor metastasis is the main cause of death of tumor patients. Approximately 90% of cancer patient deaths are due to tumor metastasis 1. Therefore, exploring the specific molecular mechanism of tumor metastasis is important content and challenge in cancer research.

Long non-coding RNA (LncRNA) is more than 200 nucleotides in length 2. It has once been treated as the simplest clone or the “noise” in transcription events. Abnormally expression of LncRNA has been shown to play important roles in regulating tumor biological behaviors, including tumor angiogenesis, drug resistance, apoptosis, and metastasis 3, 4.

Some studies reported that lncRNA LINC01296 was highly expressed in ovarian cancer (OC), hepatocellular carcinoma (HCC), thyroid cancer (TC), CRC, et al. 5-8. And, in the GEPIA database, we found that LINC01296 is highly expressed in a variety of tumors. However, the deeper complex mechanism still remains elusive. In this study, we focused on studying the interaction between LINC01296 and EMT-related molecules in tumor cells. We reported that a novel regulatory pathway composed of LINC01296/miR-141-3p/ZEB1-ZEB2 is involved in the metastasis progression of NSCLC and CRC and provides potential biomarkers and therapeutic targets for the diagnosis and treatment of tumor metastasis.

## Materials and methods

### Patients and samples

The matched lung cancer tissue (n=40) and the matched normal lung tissue (n=40) are tissue samples of patients who underwent surgery at the First Affiliated Hospital of Zhengzhou University (Zhengzhou, China) from January 2015 to November 2017. Paired colorectal cancer tissue (n=40) and matched normal bowel tissue (n=40) were obtained from patients who underwent surgery at the First Affiliated Hospital of Zhengzhou University (Zhengzhou, China) from January 2016 to December 2019 Obtained from tissue samples (Table 1). Clinicopathological parameters were collected from databases at our institutions, and all patients were followed up by phone calls every 6 months until cancer related death or study end. None of the patients received preoperative chemotherapy or radiotherapy. Through evaluating H&E tissue sections, the histological diagnosis and grade of tumor were defined on the basis of the guidelines of the World Health Organization 9. All NSCLC subjects were staged according to the International Association for the Study of Lung Cancer (IASLC) Tumor-Node-Metastasis (TNM) classification, 7th edition 10. All CRC subjects were staged according to the American Joint Committee on Cancer (AJCC) for the Study of Colorectal Cancer Tumor-Node-Metastasis (TNM) classification, 8th edition 11. The use of NSCLC and CRC tissues was approved by the Clinical Research Ethics Committee of The First Affiliated Hospital of Zhengzhou University.

### Database (DB) search

The GEPIA (Gene Expression Profile Interactive Analysis) online database (http://gepia.cancer-pku.cn) was used to analyze the expression of LINC01296 in cancers. GEPIA was also used to analyze the expression of LINC01296 between tumor and normal tissue or between different stages of each tumor. GEPIA can provide fast and customizable functions based on the Cancer Genome Atlas (TCGA) data, as well as key interactive and customizable functions, including differential expression analysis, correlation analysis and patient survival analysis 12. We used the Kaplan-Meier plotter to evaluate the prognostic value of LINC01296 in tumor patients and its correlation with ZEB1 and ZEB2 based on the data in the GEPIA online database. The binding sites of putative miRNA on lncRNA01296 sequences were predicted using StarBase V2.0 13.

### Cell culture and transfection

The human Non-small cell lung cancer cell line A549 and H1299 used in this study were obtained from the Cell Bank of the Chinese Academy of Science (Shanghai, China). The human colorectal cancer cell line SW480 and HCT116 used in this study were obtained from the Biotherapy Center of The First Affiliated Hospital of Zhengzhou University (Zhengzhou, China). The human normal colorectal mucosal cell FHC used in this study was obtained from the cell line of the Chinese Academy of Sciences (Shanghai, China). The human kidney embryo cell 293T used in this study was obtained from the cell line of the Chinese Academy of Sciences (Shanghai, China). Cells were maintained in Dulbecco's modified Eagle's medium (DMEM) (Sigma, D6429) supplemented with 10% fetal bovine serum (FBS) (Biological Industries, 04-001-1A) in a humidified incubator under a 5% CO_2_ atmosphere at 37 °C.

To explore the biological function of LINC01296, A549, H1299, SW480 ,HCT116 and FHC cells were transfected with siRNAs targeting LINC01296 (si-LINC01296) and corresponding negative controls (si-NC; RiboBio, Guangzhou, China) respectively. The miR-141-3p mimics, inhibitor and the corresponding negative controls were synthesized by RiboBio. For transfection, seed 2×10^5^ cells (per well) into a six-well plate and transfect si-LINC01296, miR-141-3p mimics or inhibitor into the cells using Lipofectamine 3000 (Thermo Fisher, L3000-015). The transfected cells were harvested after 48 to 72 hours. The transfection efficiency was determined by qPCR.

### RNA extraction and real-time PCR

Follow the manufacturer's instructions to extract total RNA from NSCLC and CRC tissues and cells using RNA iso Plus reagent (Takara, 9018). Reverse transcription of RNA was performed using Prime Script RT Master Mix Kit (Takara, RR047) according to the manufacturer's protocol. The relative quantification of LINC01296 uses the 2^-ΔΔCt^ method, with GAPDH as the internal control. MiR-141-3p expression was normalized to the internal control U6 using the ^2-ΔΔCt^ method.

### Cell migration and invasion analysis

The wound healing assay as well as the transwell assay were used to assess cell migration. In short, cells were seeded into triplicate wells of a 6-well plate and cultured to 30-50% confluence, and then a 20 μl pipette tip was used to create artificial scratches. The cell layer was imaged, and the migration was monitored at 0 and 24 hours after scratching using the CANON camera. The transwell assay was used to obtain the invasion ability of the cells. Cells (5×10^4^) were seeded on a Transwell plate with 8 mm holes, and DMEM supplemented with 20% FBS was used as a chemotactic agent. After 48 hours of incubation, use a cotton swab to manually remove non-invasive cells. Subsequently, the cells were fixed in 4% paraformaldehyde for 30 minutes, stained with crystal violet for 30 minutes, and then counted under a microscope.

### Luciferase reporter assay

A bioinformatics tool (microRNA.org) was used to predict the miR-141-3p binding site of LINC01296. LINC01296 reporter plasmid and the internal control Renilla luciferase plasmid were transfected with the appropriate plasmids using Lipofectamine 3000 (Invitrogen). The relative luciferase activity was measured 48 h after transfection, and luciferase activity was measured using a dual-luciferase reporter gene assay system (Promega, USA) according to the manufacturer's instructions.

### Western blot analysis

In short, cells were collected and lysed using RIPA protein extraction reagent (Solarbio) and protease inhibitor mixture (Solarbio). The same amount of protein was electrophoresed on an 8% SDS-PAGE gel, then transferred to a polyvinylidene fluoride (PVDF) membrane (Millipore, MA, USA), and then degreasing in a buffer (5% in TBST Blocked in milk) and then incubated with anti-rabbit ZEB1 antibody (1:1000 dilution, Cell Signaling Technology) and anti-rabbit ZEB2 antibody (1:2500 dilution, Proteintech) at 4 °C for 12 hours. Anti-rabbit GAPDH antibody (1:5000 dilution, Proteintech) was used as a loading control. Horseradish peroxidase-conjugated goat anti-rabbit or goat anti-mouse IgG antibody (1:5000, Proteintech) was used as the secondary antibody.

### Statistical analysis

All experiments were repeated three times. Statistical analysis was performed using SPSS (version 23.0, SPSS Inc.) or GraphPad Prism software (version 8.2.1, USA). The clinicopathological characteristics were analyzed by chi-square test. Kaplan Meier method and log-rank test were used to generate survival curves. According to the distribution, use Student's t test or Mann-Whitney U test to compare the two groups. P (two-sided) less than 0.05 is considered to be statistically significant. All data are expressed as mean ± standard deviation (SD).

## Results

### LINC01296 was highly expressed in cancers

**Figure 1:**
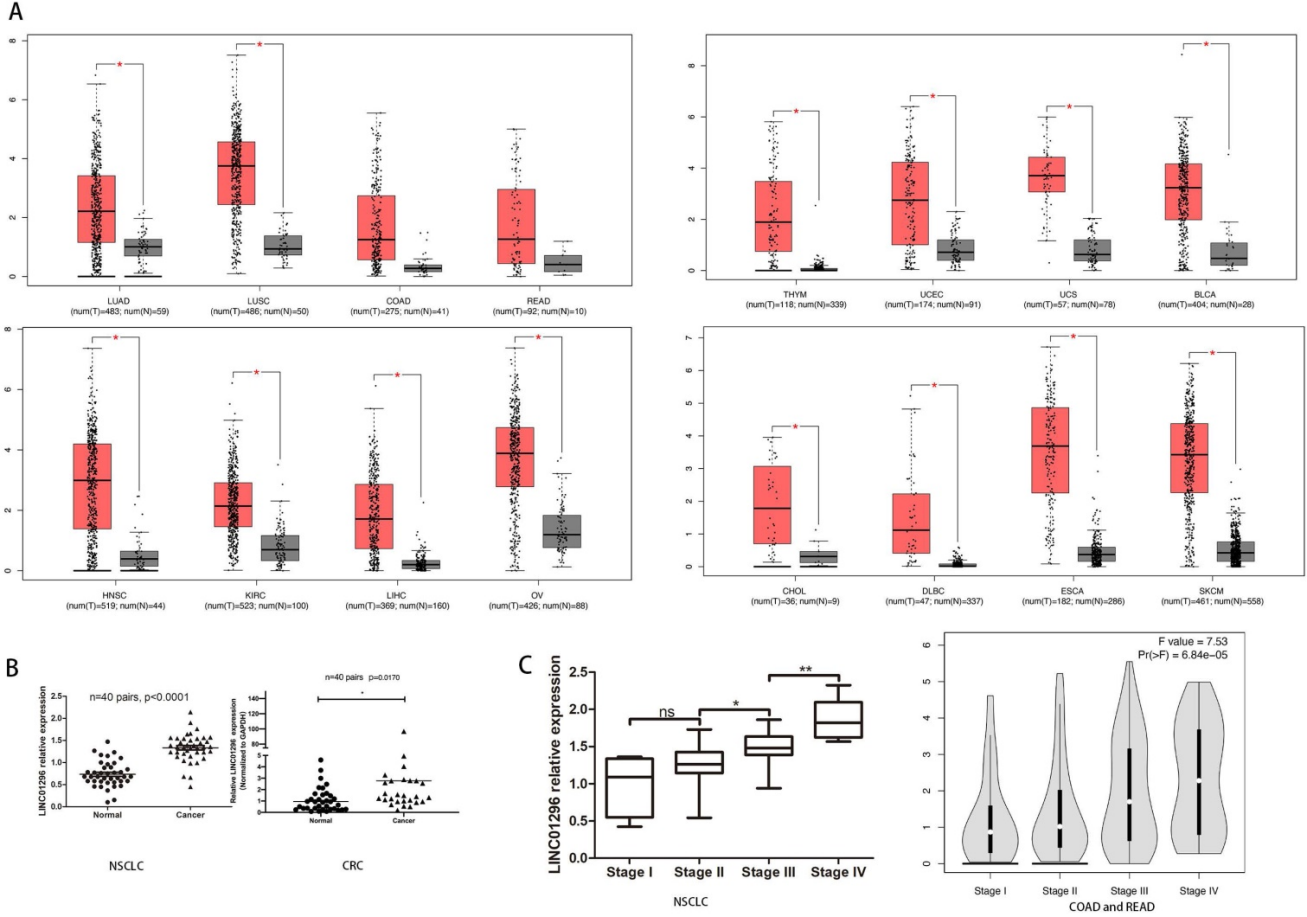
** LINC01296 was high expressed in cancers and associated with poor prognosis. (A)** LINC01296 was high expressed in different cancers tissues from GEPIA. **(B)** High LINC01296 expression was observed in NSCLC tissues and CRC tissues than corresponding adjacent normal tissue.** (C)** High LINC01296 expression was significantly associated with TNM stage in NSCLCL and CRC patients. *P < 0.05, **P < 0.01,^ ns^ P >0.05. Error bars indicate mean ± SD.

To reveal the potential role of LINC01296 in different cancers metastases, firstly, we use Gene Expression Profiling Interactive Analysis (GEPIA: http://gepia.cancer-pku.cn/index.html) to predict LINC01296 expression in different cancers tissues (Fig. 1A). Then, qRT-PCR was performed to detect the expression of LINC01296 in tumors. Elevated expression of LINC1296 was observed in NSCLC tissues than paired adjacent normal lung tissue (p<0.05), and LINC01296 expression was significantly upregulated in CRC tissues compared with nontumor tissues (P<0.05) (Fig. 1B). Moreover, high expression in NSCLC tissues was closely related with TNM stage. It is worth noting that the GEPIA database shows that the high expression of LINC01296 is related with TNM stage (p<0.05, Fig. 1C).

### LINC01296 was positively related with poor prognosis

**Figure 2:**
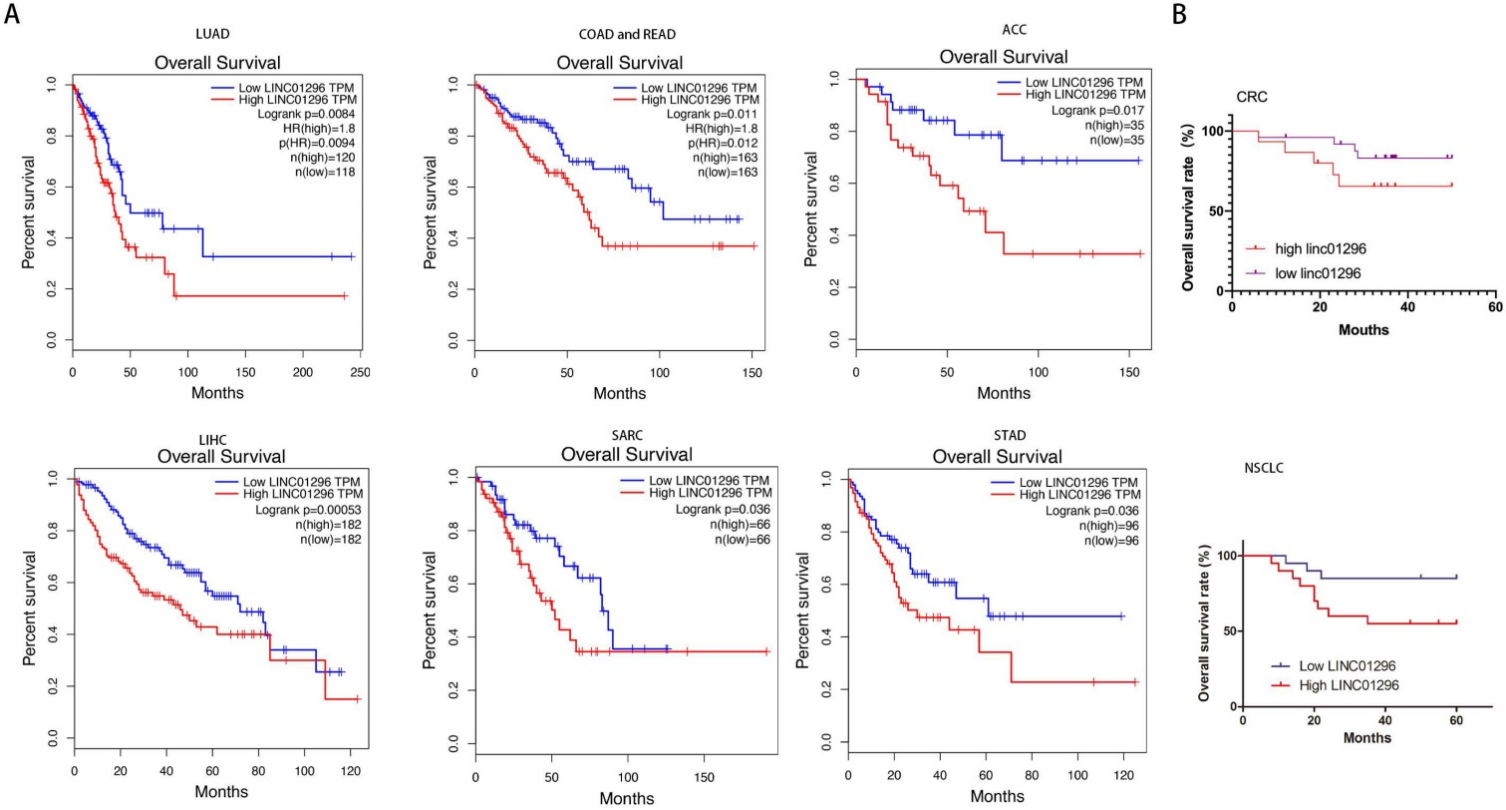
** LINC01296 was associated with poor prognosis. (A)** Survival analysis for LINC01296 expression in different tumor patients from TCGA database. High LINC01296 expression was significantly associated with poor OS in LUAD, COAD, READ, ACC, LIHC, SARC and STAD patients.** (B)** High LINC01296 expression was significantly associated with poor 5-year overall survival (OS) in 40 NSCLC and CRC patients (P<0.05).

GEPIA database with TCGA demonstrated that high LINC01296 expression in different tumors was significantly associated with poor prognosis (p<0.05, Fig. 2A). To further verify the prognostic role of LINC01296 in cancers, Kaplan-Meier analyses and log-rank tests were performed. As shown in Fig 2B, 5-year overall survival (OS) of high LINC01296 expression group was shorter than low LINC01296 expression group (p<0.05, Fig. 2B). These results indicate that highly expressed LINC01296 plays an important role in the progress of tumors.

### LINC01296 promoted tumors migration and invasion *in vitro*

**Figure 3:**
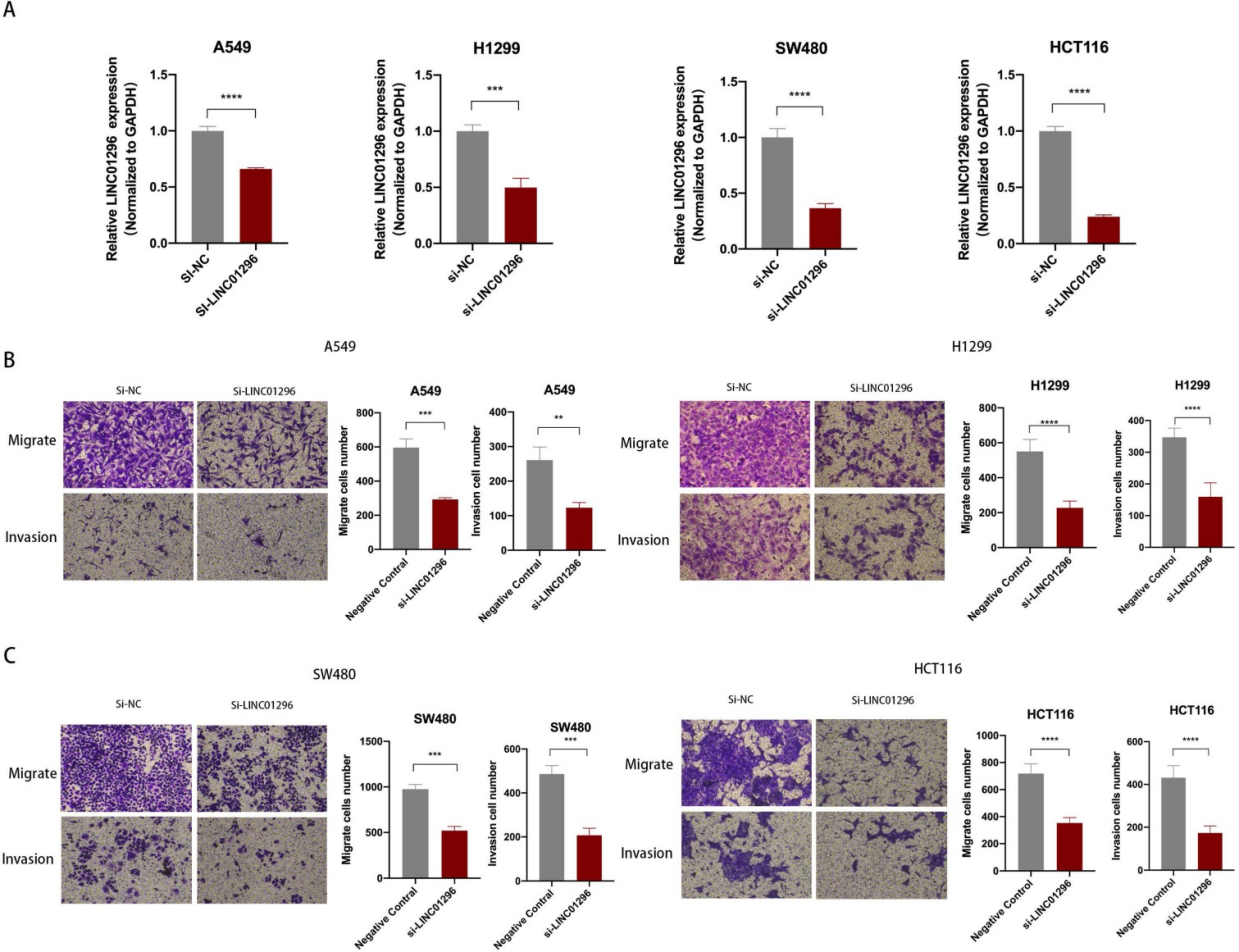
** LINC01296 promotes tumor invasion and migration. (A)** In A549, H1299, SW480 and HCT116 cells, siRNA targeting LINC01296 knocked down the expression of LINC01296.** (B)** si-LINC01296 weakened the invasion and migration of A549 and H1299 cells, as measured by Transwell analysis (magnification, ×100).** (C)** si-LINC01296 weakened the invasion and migration of SW480 and HCT116 cells, as measured by Transwell analysis (magnification, ×100). ** P <0.01, *** P <0.001, **** P <0.0001. Error bars indicate mean ± SD.

To further explore the biological role of LINC01296 in cancers, lung cancer cell lines A549, H1299 and colorectal cancer cell lines SW480, HCT116 cell lines were used in subsequent experiments. We next transfected A549, H1299, SW480 and HCT116 cells with LINC01296-siRNA (si-LINC01296) and the negative control (si-NC). The transfection efficiency was confirmed by qPCR (Fig. 3A). To investigate whether LINC01296 plays an important role in the pathogenesis of tumor metastasis, Wound healing and transwell assays were performed respectively. The results of the Wound-healing assay demonstrated that the LINC01296 knockdown significantly inhibited cell migration (Fig. S1A). Transwell assays demonstrated that LINC01296 knockdown inhibited cell invasion and migration (Fig. 3B, C). These results indicated the potential carcinogenicity of LINC01296 in cancers.

### LINC01296 acted as a molecular sponge for miR-141-3p in tumors

**Figure 4:**
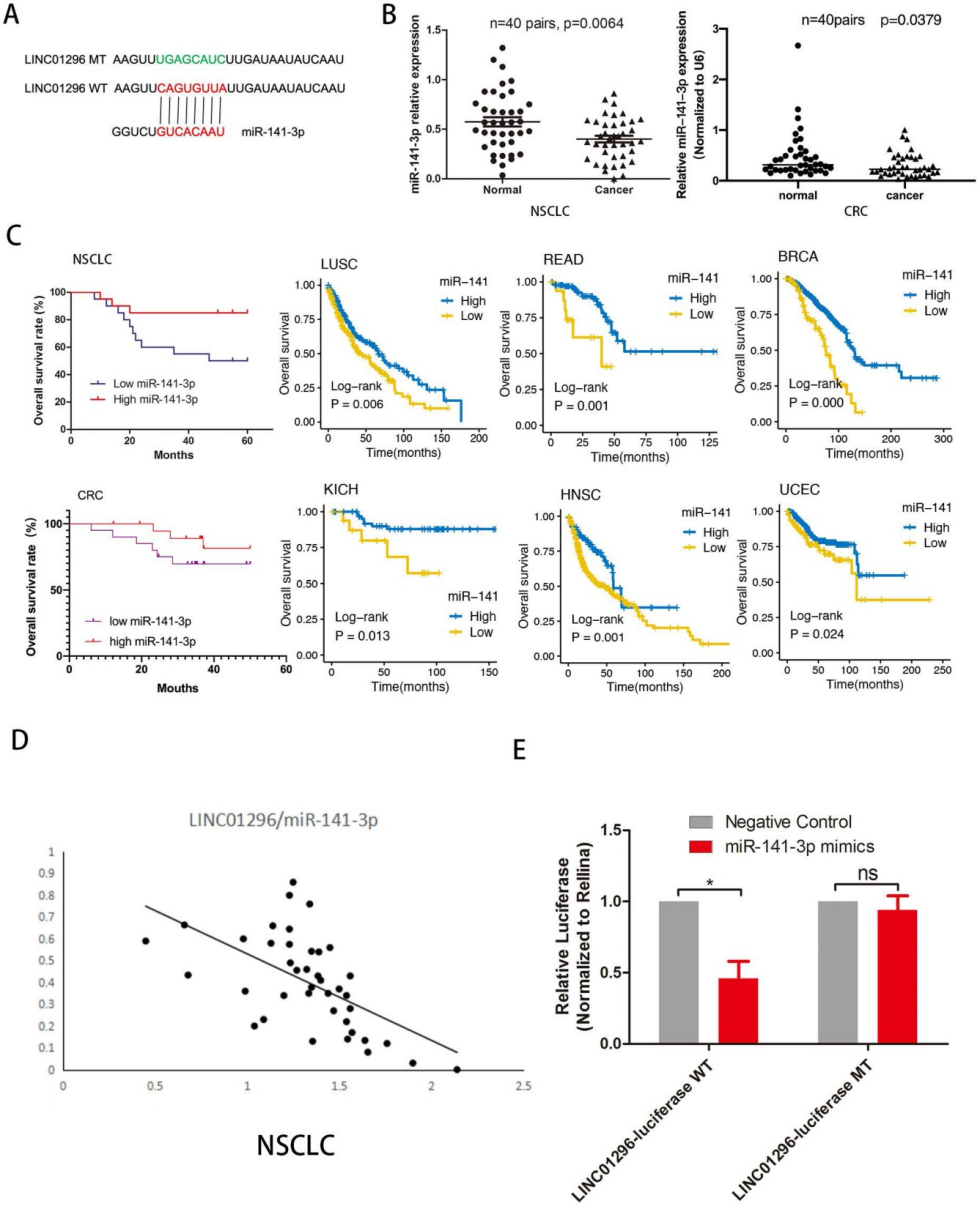
** LINC01296 sponged miR-141-3p as ceRNA in cancers. (A)** The schematic of miR-141-3p and LINC01296 3'UTR or mutant sequences.** (B)** qRT-PCR was performed to detect the expression of miR-141-3p in NSCLC tissues and CRC tissues. The results showed that miR-141-3p was down-regulated in NSCLC and CRC tissues **(C)** Kaplan-Meier analysis and log-rank test were performed to analyze OS in high and low miR-141-3p expression NSCLC and CRC patients. Low miR-141-3p expression was significantly associated with poor OS in NSCLC and CRC patients. Survival rate of miR-141-3p in different tumor types in the TCGA database. Low miR-141-3p expression was significantly associated with poor OS in LUSC, READ, BRCA, KICH, HNSC and UCEC patients. **(D)**Pearson correlation analysis was conducted to analysis the relationship between LINC01296 and miR-141-3p level was measured in 40 NSCLC tissues using. LINC01296 expression and miR-141-3p expression were inversely correlated in NSCLC tissues. **(E)**Luciferase assays showed that miR-141-3p reduced the luciferase activity of LINC01296-WT in 293T cells (p<0.05).

To investigate whether LINC01296 plays a similar role in different cancers, we predicted miRNA target sites using the online microRNA-target program (http://www.microRNA.org) and found out miR-141-3p with relevant binding sites in LINC01296 (Fig. 4A). The expression level of miR-141-3p was measured in NSCLC and CRC tissues and paired adjacent normal adjacent tissues by qRT-PCR, demonstrating that miR-141-3p was lower expressed in NSCLC and CRC tissues than normal adjacent tissues (p<0.05, Fig. 4B). Furthermore, Kaplan-Meier analyses and log-rank test in 40 NSCLC and 40 CRC cases demonstrated that 5-year overall survival (OS) was longer in high miR-141-3p group than low miR-141-3p group. It is worth noting that the TCGA database shows that low expression of miR-141-3p has a lower survival rate in a variety of cancer types (P<0.05, Fig. 4C). Interestingly, miR-141-3p level was significantly negatively correlated with LINC01296 level. In addition, the TCGA database shows that the expression of LINC01296 is negatively correlated with miR-141-3p in a variety of cancer types (Fig. 4D and Fig. S2). Dual-luciferase reporter assay revealed that over-expression of miR-141-3p reduced the luciferase activity of the pMIR luciferase reporter containing LINC01296-WT, but not the reporter containing LINC01296-MT (Fig. 4E).

### miR-141-3p regulated EMT by regulating ZEB1 and ZEB2 expression

**Figure 5:**
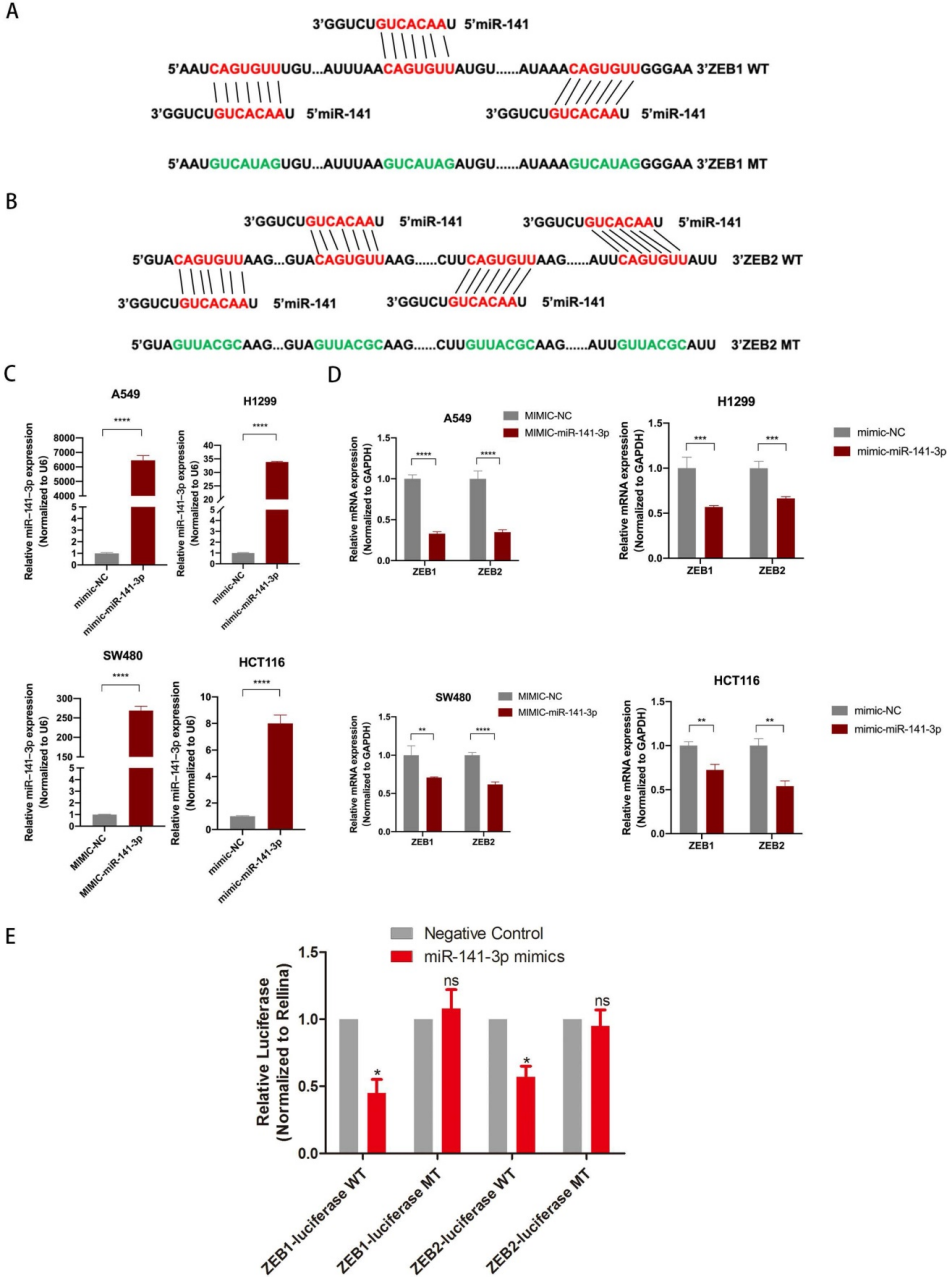
** miR-141-3p inhibit the expression of ZEB1 and ZEB2. (A, B)** The schematic of miR-141-3p and LINC01296 3'UTR or mutant sequences. **(C)** Knockdown of miR-141-3p in A549, H1299, SW480 and HCT116 cells was analyzed by qRT-PCR. **(D)** Analysis of ZEB1 and ZEB2 expression levels in A549, H1299, SW480 and HCT116 cells transfected with miR-141-3p mimic by RT-qPCR. **(E)** Luciferase assays showed that miR-141-3p reduced the luciferase activity of ZEB1-WT and ZEB2-WT in 293T cells. * P <0.05, ** P <0.01, *** P <0.001, **** P <0.0001,^ ns^ P >0.05. Error bars indicate mean ± SD.

To further explore the downstream target genes of miR-141-3p, we used TargetScan to predict the target genes of miR-141-3p. As we all know, the relationship between EMT-related molecules and tumor metastasis is inseparable. ZEB1 and ZEB2 were selected. ZEB1 and ZEB2 were well-accepted oncogene targeted by miR-141-3p in tumors 14, 15. Bioinformatics software (microRNA) to predict the potential miR-141-3p binding sites in ZEB1 and ZEB2 (Fig. 5A,B). The epithelial-mesenchymal transition (EMT) process is important for tumor cell invasion and metastasis. Interestingly, the TCGA database showed that in a variety of tumors, the expression of miR-141-3p and ZEB1 showed a negative correlation trend. Similarly, the expression of miR-141-3p and ZEB2 in a variety of tumors showed a negative correlation trend (Fig. S3). To clarify the relationship between ZEB1, ZEB2 and miR-141-3p, we next transfected A549, H1299, SW480 and HCT116 cells with miR-141-3p-mimics (mimic-miR-141-3p) and the negative control (mimic-NC). The transfection efficiency was confirmed by qPCR (Fig. 5C). In addition, in order to ensure the specificity of our experimental results, we have also transfected mimic-NC or mimic-miR-141-3p into the normal intestinal epithelial cell FHC cell line (Fig. S1 C). As suggested by Fig. 5D, qPCR showed that high expression of miR-141-3p down-regulated the expression of ZEB1 and ZEB2 (Fig. 5D). But in the FHC cell line, the expression of ZEB1 and ZEB2 did not change significantly (Fig. S1C). Then, Dual-luciferase reporter assay revealed that over-expression of ZEB1 and ZEB2 reduced the luciferase activity of the pMIR luciferase reporter containing miR-141-3p-WT, but not the reporter containing miR-141-3p -MT (Fig. 5E).

### LINC01296 regulated EMT-related molecular expression by targeting miR-141-3p

**Figure 6:**
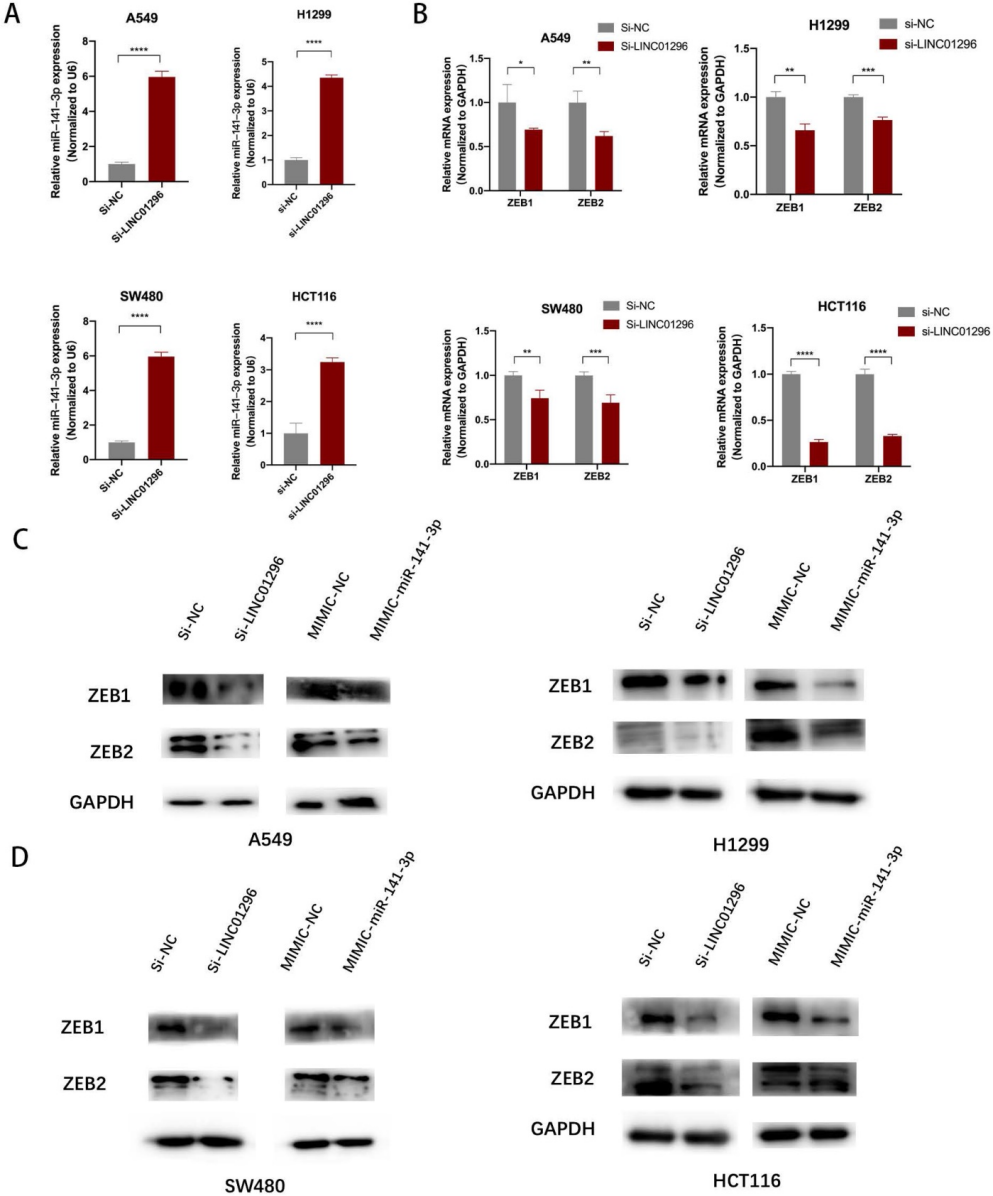
** LINC01296 regulated EMT- related molecular expression by targeting miR-141-3p. (A)** LINC01296 knockdown regulates miR-141-3p expression level in A549, H1299, SW480 and HCT116 cells. **(B)** si-LINC01296, inhibitor control, miR-141-3p mimics, mimics control was transfected into A549, H1299, SW480 and HCT116 cells, ZEB1/ZEB2 were detected by QT-PCR assay to confirm the effect of LINC01296 and miR-141-3p on the above two molecules. **(C)** Protein levels of ZEB1 and ZEB2 in A549 and H1299 cells transfected with the si-LINC01296 and miR-141-3p mimic were determined by western blot. **(D)** Protein levels of ZEB1 and ZEB2 in SW480 and HCT116 cells transfected with the si-LINC01296 and miR-141-3p mimic were determined by western blot. * P <0.05, ** P <0.01, *** P <0.001, **** P <0.0001. Error bars indicate mean ± SD.

TCGA database shows that LINC01296 level was significantly positively correlated with ZEB1 and ZEB2 levels in different cancers (Fig. S4). To make clear the relationship between LINC01296, miR-141-3p, and ZEB1/ZEB2, the siRNA-LINC01296 were transfected into A549, H1299, SW480 and HCT116 cells. The results showed that the low expression of LINC01296 would promote the expression of miR-141-3p (*p*<0.05, Fig. 6A). We used miR-141-3p mimics to transfect A549, H1299, SW480 and HCT116 cells, as suggested by Fig. 5D, qPCR showed that high expression of miR-141-3p down-regulated the expression of ZEB1 and ZEB2 (Fig. 5D). And we found that after knocking down LINC01296, the expression of ZEB1 also decreased (*p*<0.05, Fig. 6B). Simultaneously, ZEB2 expression was lower in si-LINC01296 group than inhibitor control group (*p*<0.05, Fig. 6B). In addition, in order to ensure the specificity of our experimental results, we have also transfected si-NC or si-LINC01296 into the normal intestinal epithelial cell FHC cell line (Fig. S1B), the results showed that after knocking down LINC01296 in the FHC cell line, the expression of miR-141-3p did not change significantly, and the expression of ZEB1 and ZEB2 also did not change significantly (Fig. S1B).

Western blotting was performed to detect EMT-related molecule levels. The results demonstrated that LINC01296 knockdown downregulated ZEB1, ZEB2 compared to negative controls in A549, H1299, SW480 and HCT116 cells. Additionally, ZEB1, ZEB2 were downregulated in miR-141-3p mimics group compared to negative controls group (Fig. 6C, D).

However, in FHC cell lines transfected with si-LINC01296 or mimic-miR-141-3p, western blot showed that the expression of ZEB1 and ZEB2 did not change significantly compared with the corresponding control group (Fig. S1D). These results suggest that LINC01296 regulates EMT-related molecular expression by targeting miR-141-3p *in vitro*.

### The restoration of ZEB1 and ZEB2 reversed the inhibition of migration and invasion of SW480 and A549 cells induced by LINC01296 knockdown

**Figure 7:**
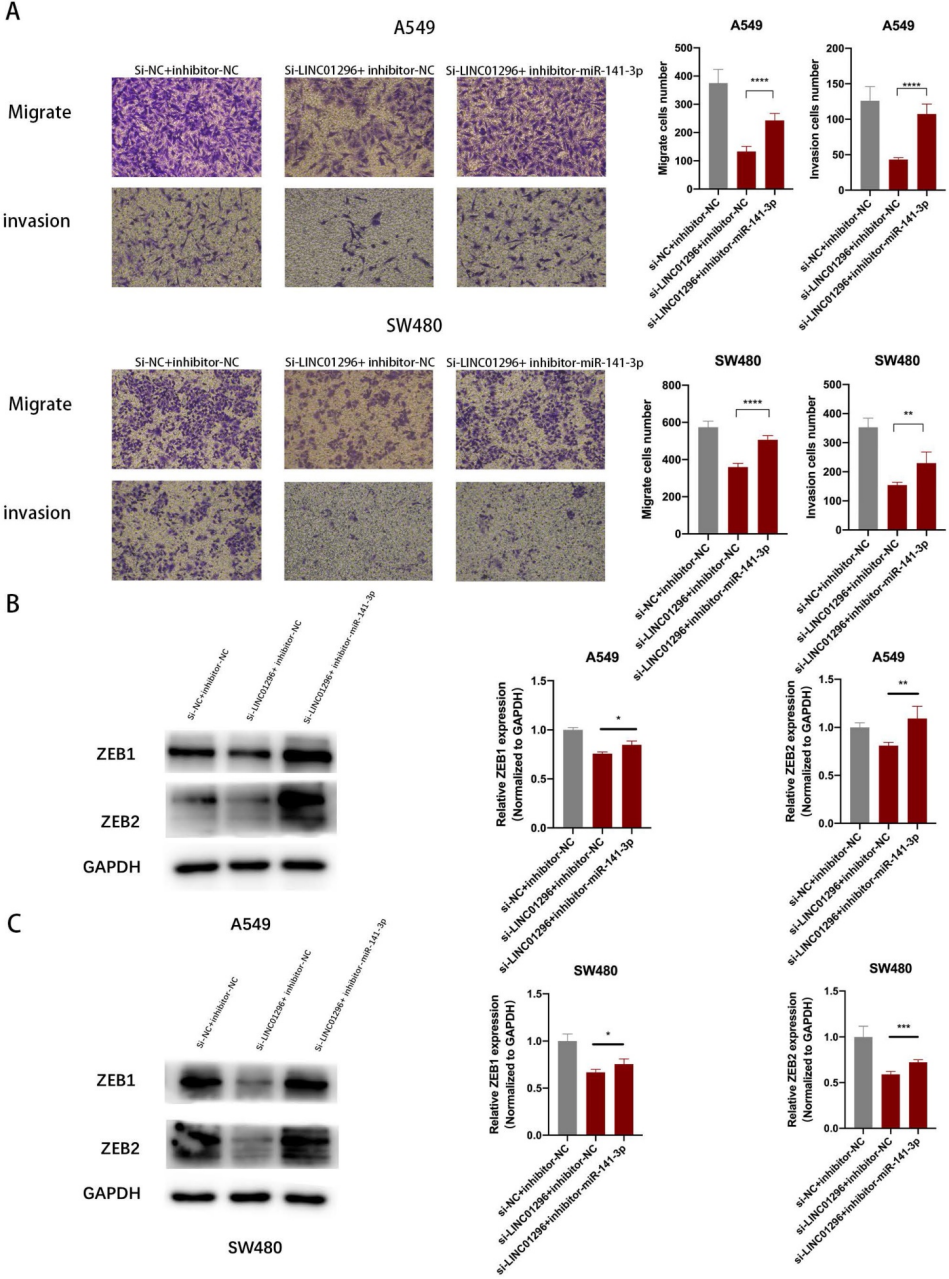
** The restoration of ZEB1 and ZEB2 reversed the inhibition of migration and invasion of SW480 and A549 cells induced by LINC01296 knockdown. (A)** After miR-141-3p inhibitor was introduced into SW480 and A549 cells, the cell invasion and migration ability induced by LINC01296 was significantly enhanced, which was measured by Transwell assay (magnification, × 100). **(B)** The effect of si-LINC01296, miR-141-3p inhibitor and si-LINC01296 + miR141-3p inhibitor on the expression levels of ZEB1 and ZEB2 in A549 cells. **(C)** The effect of si-LINC01296, miR-141-3p inhibitor and si-LINC01296 + miR141-3p inhibitor on the expression levels of ZEB1 and ZEB2 in SW480 cells. * P <0.05, ** P <0.01, *** P <0.001, **** P <0.0001. Error bars indicate mean ± SD.

To further investigate whether the regulation of LINC01296 on the migration and invasion of SW480 and A549 cells depends on miR-141-3p, and to further verify that LINC01296 can regulate the expression of ZEB1/ZEB2 by targeting miR-141-3p in tumor cells, first we co-transfect si-LINC01296 and miR-141-3p inhibitor (inhibitor-miR-141-3p) into SW480 and A549 cell lines and verify cell migration and invasion by transwell analysis. We found that in the group co-transfected with si-LINC01296 and miR-141-3p inhibitors, the regulation of LINC01296 on tumor cell migration and invasion was reversed (Fig. 7A). We further verified whether the regulation of ZEB1 and ZEB2 by LINC01296 depends on miR-141-3p through Western blotting and qRT-PCR. We found that in the group co-transfected with si-LINC01296 and miR-141-3p inhibitor, the regulatory effect of LINC01296 on ZEB1 and ZEB2 expression were reversed (Fig. 7B, C). The above results suggested that LINC01296 regulated ZEB1 and ZEB2 expression by sponging miR-141-3p and the restoration of ZEB1 and ZEB2 reversed the inhibition of migration and invasion of SW480 and A549 cells induced by LINC01296 knockdown.

## Discussion

LncRNA has been shown to have a negative or positive effect on the expression of the coding gene through a variety of mechanisms, such as chromatin remodeling, encoding protein, etc. 16. At present, the key functions of lncRNAs have been involved in regulating almost all physiological and pathological processes, through multiple unknown regulatory mechanisms, such as cell growth, autophagy and development, cell cycle 3. LncRNAs also play essential roles drivers of tumor suppressive and oncogenic functions and constitute an important component of cancer biology 17, 18. For example, a maternally expressed imprinted lncRNA, H19, acts as an endogenous sponge of miRNA let-7, which can directly bind to let-7 and regulate the expression of multiple genes involved in EMT, such as ZEB1 and ZEB2 19.

In this study, LINC01296 was identified to be significantly upregulated in NSCLC and CRC patient tissues and high LINC1296 expression in tumors was significantly associated with poor overall survival. In addition, the TCGA database shows that high expression of LINC01296 is associated with poor survival in a variety of tumors. In accordance with this, overexpression of LINC01296 remarkably promoted migration and invasion *in vitro*. Interestingly, the putative miRNA binding sites on LINC01296 sequences were predicted using StarBase V2.0, showing that LINC01296 has the potential to target miR-141-3p.

MiRNA has been widely reported to have an important role in EMT and regulate the expression of EMT-related genes 20. EMT is the process of converting epithelial cells into active mesenchymal cells, which results in invasive and migratory ability 21. The role of EMT in tumor proliferation, autophagy, and metastasis has been verified in multiple ways. The present study concluded that LINC01296 facilitates EMT in NSCLC and CRC cell lines. Currently, the well-known EMT regulatory proteins include ZEB1, ZEB2 22, 23. In this study, Bioinformatics software (microRNA) was used to predict the potential miR-141-3p binding sites in ZEB1 and ZEB2, then Dual-luciferase reporter assay verified the relationship between miR-141-3p and ZEB1/ZEB2. Consequently, this revealed that miR-141-3p inhibits EMT in NSCLC and CRC by regulating the expression of ZEB1 and ZEB2.

In order to verify the molecular mechanism of LINC01296 regulating EMT by targeting miR-141-3p, we conducted further studies. Studies have concluded that lncRNA can adsorb miRNA of certain target genes in the cytoplasm by the so-called 'sponge effect' 24. This can result in inhibition of miRNA on specific target genes, which indirectly promoting the expression of target genes at the post-transcriptional level. MicroRNA (miRNA) is an endogenous, small RNA with a length approximating 20-24 nucleotides, and has multiple important regulatory roles in cells 25. Long noncoding RNA LINC01296 harbors miR-141-3p to regulate NSCLC and CRC migration and invasion. StarBase V2.0 predicts that LINC01296 has a binding site with miR-141-3p based on its molecular structure. Through transfection experiments, we revealed that LINC01296 can indeed regulate the expression of EMT-related molecule through targeting miR-141-3p. Luciferase experiments were performed to clarify the relationship between LINC01296 and miR-141-3p, which were in accordance with our predictions. These results concluded that LINC01296 can bind miR-141-3p through a sponge effect, thus upregulating the expression of EMT-related molecule, such as ZEB1, ZEB2.

Taken together, the present results outline a possible role and mechanism of LINC01296 in regulating metastasis of lung adenocarcinoma by targeting miR-141-3p, promoting EMT and EMT-related molecule expression. These findings indicated that LINC01296 is an important potential marker for predicting prognosis, and an important target for tumors therapy.

## Supplementary Material

Supplementary figures and table.

## Figures and Tables

**Table 1:** Clinicopathological characteristics of 40 patients with lung cancer and 40 patients with colorectal cancer

Lung cancer			Colorectal cancer		
Characteristics		N	Characteristics		N
Tumor/Adjacent	Tumor	40	Tumor/Adjacent	Tumor	40
	Adjacent	40		Adjacent	40
Age	≤61	20	Age	≤61	19
	>61	20		>61	21
Sex	Male	28	Sex	Male	19
	Female	12		Female	21
Tissue type	Squamous cell carcinoma	21	Tissue type	Rectal cancer	25
	Adenocarcinoma	14		Colon cancer	15
	other	5			
Stage	1	4	Stage	1	0
	2	12		2	10
	3	19		3	27
	4	5		4	3

## Abbreviations

lncRNAs: Long noncoding RNAs
CRC: colorectal cancer
LAML: Acute Myeloid Leukemia
UCS: Uterine Carcinosarcoma
KIRC: Kidney renal clear cell carcinoma
THCA: Thyroid carcinoma
SKCM: Skin Cutaneous Melanoma
UVM: Uveal Melanoma
EMT: epithelial-mesenchymal transition
CD: Crohn's disease
IBD: inflammatory bowel disease
OS: overall survival
DFS: disease free survival
