# Supplementary material for: LINC01296/miR-141-3p/ZEB1-ZEB2 axis promotes tumor metastasis via enhancing epithelial-mesenchymal transition process
Source: J Cancer. 2021 Mar 5;12(9):2723–34. doi: 10.7150/jca.55626 (PMC8040730; doi:10.7150/jca.55626)
Supplement: Supplementary file 1 — Supplementary figures and table. [file jcav12p2723s1.pdf]

Supplementary figure 1

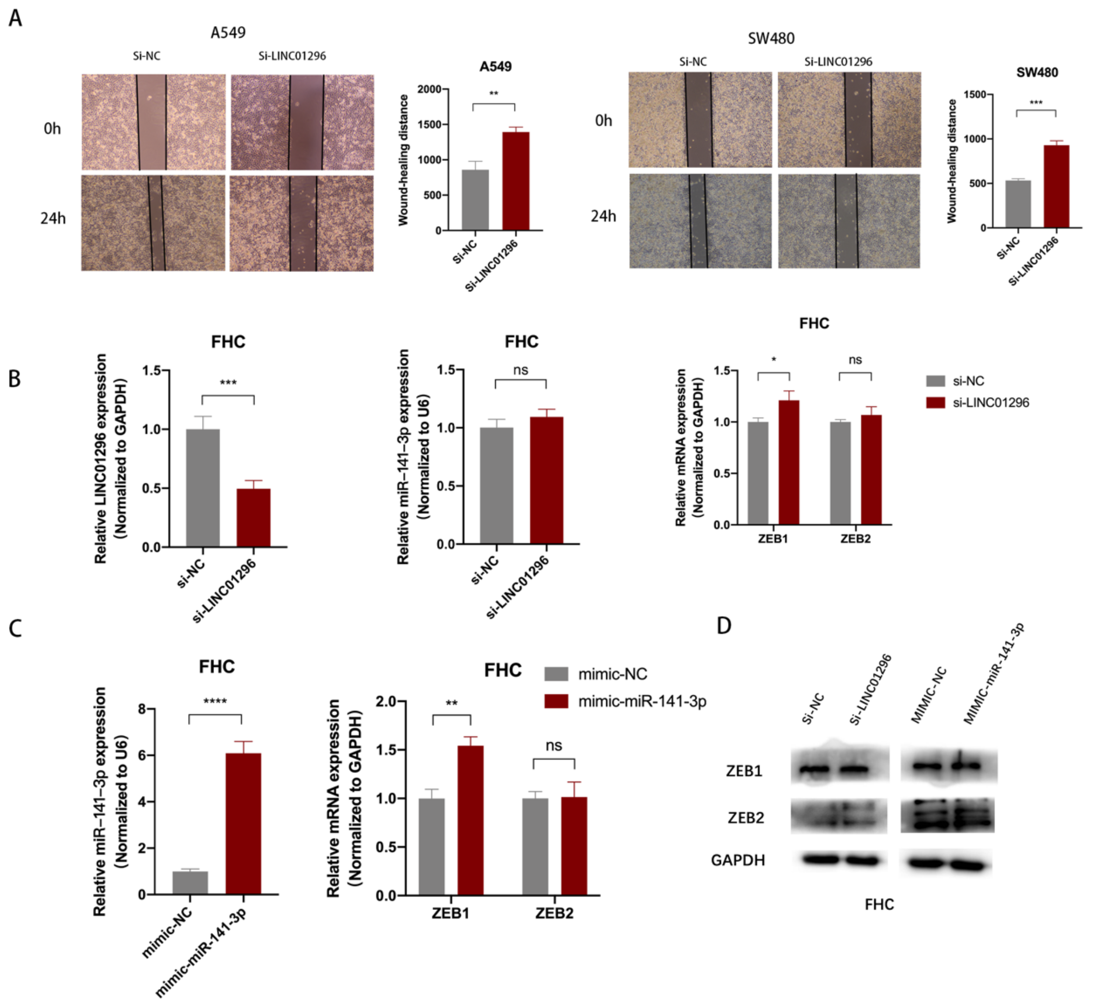

Figure S1: (A) si-LINC01296 weakened the migration of A549 and SW480 cells, as measured by Scale bar, 100μm and wound healing test. **(B)** Transfect FHC cells with si-NC or si-LINC01296. qRT-PCR showed that si-LINC-1296 down-regulated the expression of LINC01296, but not up-regulated the expression of miR-141-3p and had no effect on the expression of ZEB1 and ZEB2. **(C)** Transfect FHC cells with mimic-NC or mimic-miR-141-3p. qRT-PCR showed that mimic-miR-141-3p up-regulated the expression of miR-141-3p, but not down-regulated the expression of ZEB1 and ZEB2. **(D)** Transfect FHC cells with si-1296 or mimic-miR-141-3p. Western blot showed that si-LINC-1296 or mimic-miR-141-3p did not regulate the expression of ZEB1 and ZEB2.

Supplementary figure 2

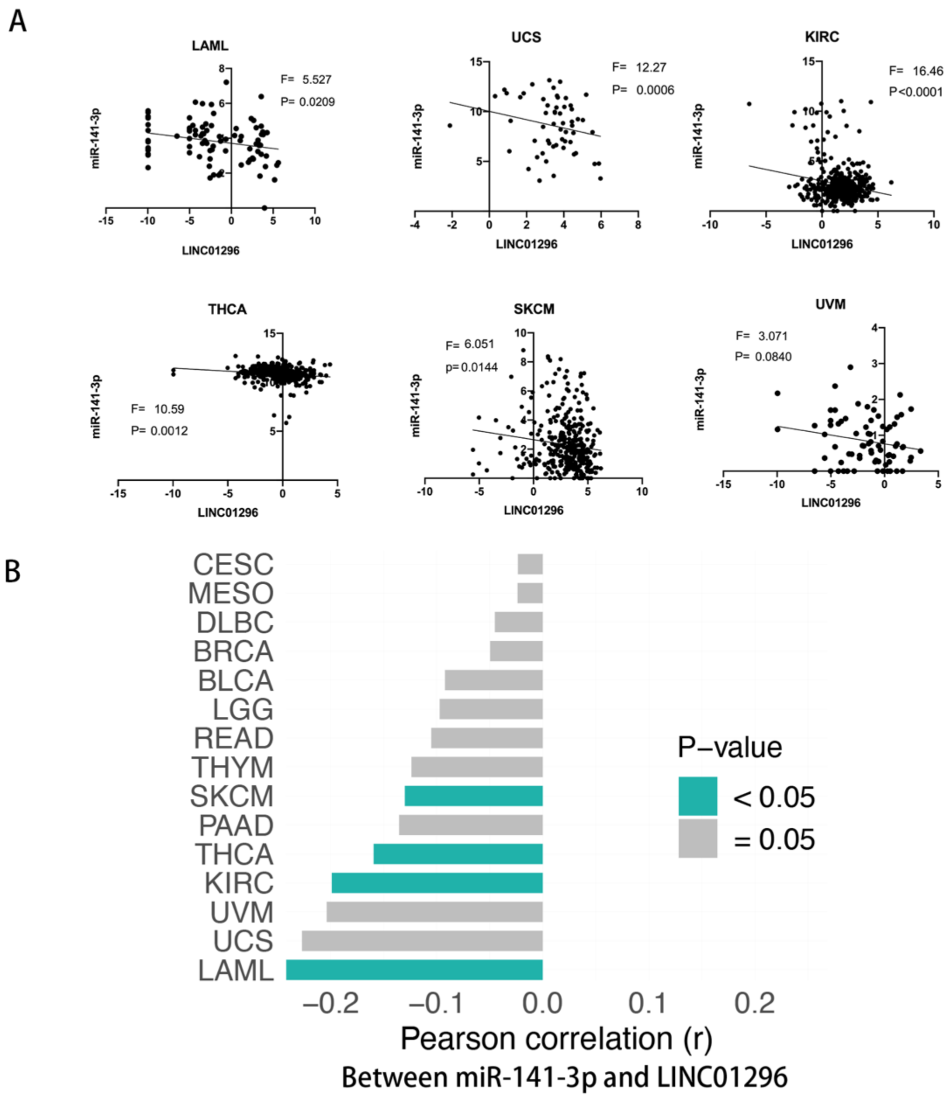

Figure S2 Correlation analysis of LINC01296 and miR-141-3p in various tumors. (A) In the TCGA database, LINC01296 and miR-141-3p show a negative correlation in LAML, UCS, KIRC, THCA, SKCM and UVM. **(B)** In the TCGA database, LINC01296 and miR-141-3p show a negative correlation trend in different tumors.

Supplementary figure 3

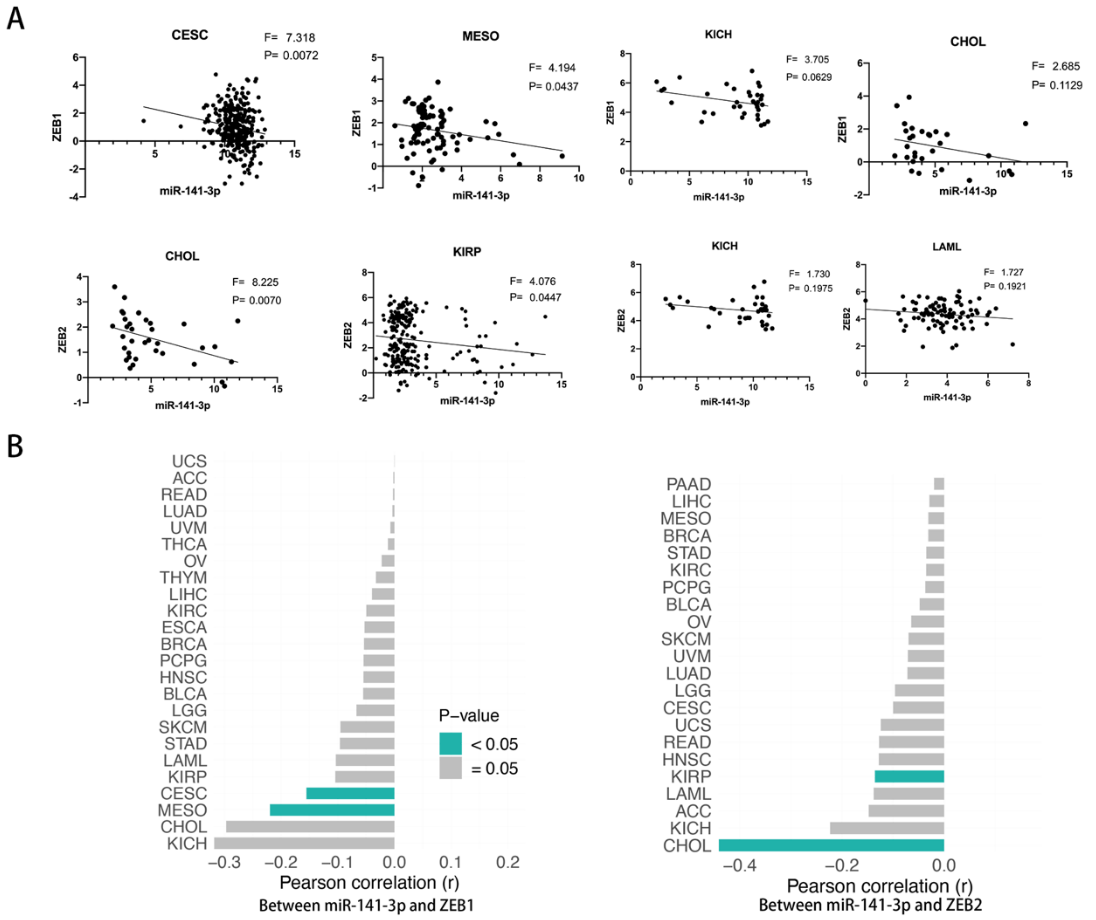

Figure S3 Correlation analysis of TCGA database miR-141-3p and ZEB1/ZEB2 in various tumors.

(A) Correlation analysis of TCGA database shows that miR-141-3p and ZEB1/ZEB2 in CESC, MESO, KICH, CHOL, KIRP, KICH and LAML. **(B)** Correlation analysis of TCGA database shows that miR-141-3p and ZEB1/ZEB2 in different tumors.

Supplementary figure 4

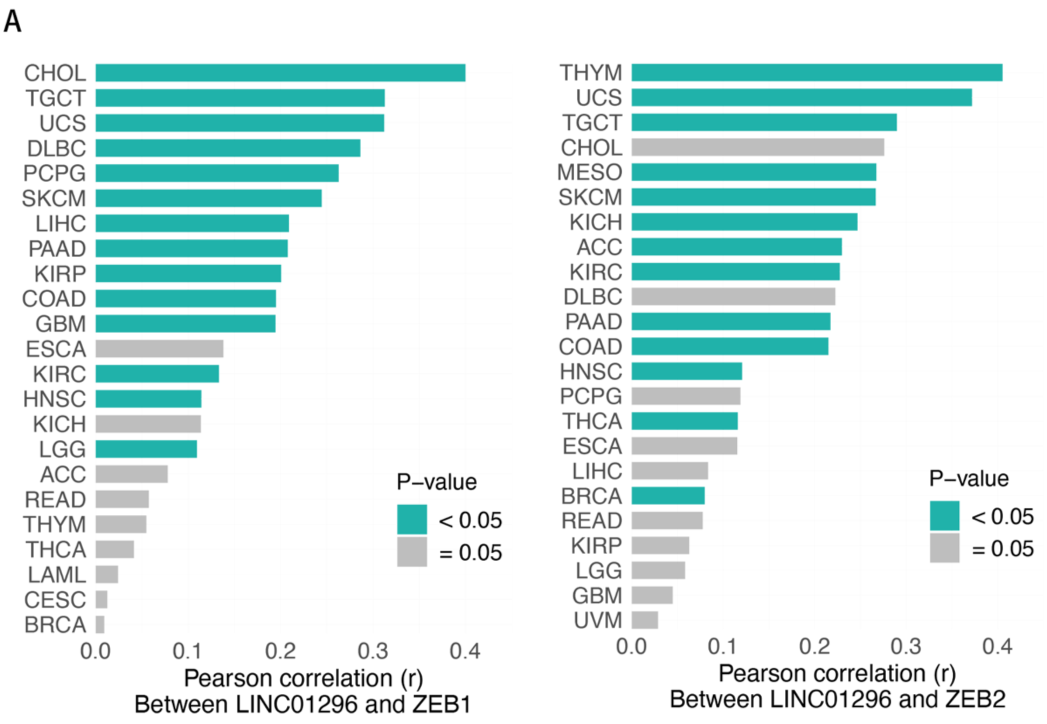

Figure S4 Correlation analysis of TCGA database LINC01296 and ZEB1/ZEB2 in various tumors.

(A) TCGA database shows that LINC01296 and ZEB1/ZEB2 are positively correlated in a variety of cancers.

Supplementary table

Table S1 The forward and reverse primers for qRT-PCR

Genes	Forward (5'- to 3'-)	Reverse (5'- to 3'-)
LINC01296	AACTGGCACCAGCCTCACT	CGGCCAACTTCTTTACCATC
hsa-miR-141-3p	CCGCGCTAACACTGTCTGGTAA	AGTGCAGGGTCCGAGGTATT
ZEB1	GCAGAUACUACACCAACUCTT	AUGGUGGUUAGUCAGUUGCTT
ZEB2	AACACCCCTGGCACAACAAC	GCTGACTGCATGACCATCGC
U6	GCTTCGGCAGCACATATACT	GTGCAGGGTCCGAGGTATTC
GAPDH	GGAGCGAGATCCCTCCAAAAT	GGCTGTTGTCATACTTCTCATGG
